# Contrast-Enhanced Mammography Versus Digital Mammography and Tomosynthesis in Dense Breasts: Diagnostic Accuracy and Impact on Surgical Planning in Multifocal and Multicentric Breast Carcinoma

**DOI:** 10.7759/cureus.99074

**Published:** 2025-12-12

**Authors:** Mariam Malik, Umal Baneen Zahra, Mariam Fayyaz, Rana Bilal Idrees, Imran Abdullah, Zeeshan Rashid Mirza, Muhammad Aasim

**Affiliations:** 1 Radiology, Institute of Nuclear Medicine and Oncology (INMOL) Cancer Hospital, Lahore, PAK; 2 Oncology, Institute of Nuclear Medicine and Oncology (INMOL) Cancer Hospital, Lahore, PAK; 3 Diagnostic Radiology, Institute of Nuclear Medicine and Oncology (INMOL) Cancer Hospital, Lahore, PAK; 4 Data Management, National Health Research Complex (NHRC) Research Centre of Health Research Institute-National Institutes of Health (HRI-NIH), Lahore, PAK

**Keywords:** contrast-enhanced mammography, dense breasts, digital breast tomosynthesis, histopathology correlation, multifocal breast cancer, surgical planning

## Abstract

Background: Dense breast tissue poses a major diagnostic challenge in breast cancer detection, as it can obscure lesions on digital mammography (DM) and limit the sensitivity of digital breast tomosynthesis (DBT). Contrast-enhanced mammography (CEM) combines morphological and functional assessment, potentially improving detection accuracy and influencing surgical decision-making.

Purpose: To compare the diagnostic performance of CEM, DM, and DBT in detecting multifocal and multicentric breast cancer in women with dense breasts, using histopathology as the reference standard, and to evaluate the impact of CEM findings on surgical management.

Materials and methods: This prospective comparative study included 185 women with Breast Imaging Reporting and Data System (BI-RADS) density categories C or D who underwent DM, DBT, and CEM prior to biopsy or surgery between April and June 2025. Two experienced breast radiologists independently evaluated lesion detection, conspicuity, diagnostic confidence, and lesion size. Statistical analyses included McNemar's test, Wilcoxon signed-rank test, Bland-Altman analysis, Pearson correlation, intraclass correlation coefficient (ICC), and logistic regression to assess predictors of surgical change.

Results: CEM detected the index lesion in 96.8% of cases, outperforming DBT (69.2%) and DM (51.9%) (p < 0.001). Lesion conspicuity and radiologist confidence were highest with CEM (p < 0.001). CEM identified ≥2 additional malignant lesions in 43.7% of patients, closely matching histopathology (43.2%), and altered surgical management in 36.8% of cases--prompting wider excision (16.8%) or mastectomy (16.2%). Lesion size correlation with histopathology was strongest for CEM (r = 0.969-0.987; ICC > 0.87). Logistic regression revealed that additional CEM-detected lesions were the strongest independent predictor of surgical modification (OR ≈ 71.7, p < 0.001). Bland-Altman plots confirmed excellent agreement with minor overestimation (mean bias ≈ +5 mm).

Conclusion: CEM demonstrated superior diagnostic performance over DM and DBT in women with dense breasts, offering enhanced lesion conspicuity, greater radiologist confidence, and high concordance with histopathology. Its significant impact on surgical planning highlights CEM as a cost-effective, accessible alternative to MRI for preoperative assessment in dense-breast populations, particularly in resource-limited settings.

## Introduction

Breast cancer remains the most commonly diagnosed cancer among women worldwide [1,2] and a leading cause of cancer-related mortality [3]. In Asia, Pakistan holds the highest incidence of breast cancer, with one in every nine Pakistani women estimated to develop breast cancer during her lifetime [4]. Early and accurate detection plays a crucial role in improving prognosis, guiding appropriate treatment, and reducing mortality. However, breast cancer detection remains particularly challenging in women with dense breast tissue (Breast Imaging Reporting and Data System (BI-RADS) density categories C and D). Dense breasts not only increase the risk of developing breast cancer [5] but also limit the diagnostic performance of conventional imaging, particularly digital mammography (DM), by masking lesions and reducing sensitivity [6]. Digital breast tomosynthesis (DBT), also known as 3D mammography, has shown promising results in improving lesion detection by reducing the effect of overlapping breast tissue. Nevertheless, DBT also has limitations [7], especially in detecting subtle lesions or evaluating the full extent of multifocal and multicentric disease in dense breasts. 

Contrast-enhanced mammography (CEM), a newer imaging technique, combines morphological and functional imaging by highlighting areas of increased vascularity through the use of iodinated contrast agents. CEM has demonstrated improved detection rates for breast cancer, particularly in dense breasts [8], and shows potential for better identification of additional cancer foci that may not be visualized on DM or DBT [9]. Despite these advantages, there is limited data comparing the diagnostic performance of CEM with DM and DBT in detecting multifocal and multicentric breast cancer, specifically in women with dense breasts, using histopathology as the gold standard.

The primary objective of this study is to compare the diagnostic performance of contrast-enhanced mammography (CEM), digital mammography (DM), and digital breast tomosynthesis (DBT) for detecting multifocal or multicentric breast cancer in women with dense breasts, using histopathology as the reference standard. The study cohort is intentionally enriched for patients with dense breasts and clinically or radiologically suspected multifocal or multicentric disease in the affected breast. This targeted inclusion is designed to assess the diagnostic utility of each modality in complex, lesion-rich settings rather than in a routine dense-breast screening population. The secondary objectives of the study are to assess (i) agreement between imaging and histopathologic lesion size (intraclass correlation coefficient), (ii) lesion conspicuity and reader confidence scores across modalities. The exploratory objective is to assess the impact of CEM on surgical planning by identifying additional lesions not seen on DM or DBT, hence identifying imaging predictors of surgical plan modification in the subset of patients with complete surgical data. This study tests the hypothesis that CEM would outperform DM and DBT in detecting additional malignant foci in dense breasts, thereby influencing surgical decision-making. Of the 185 patients included, complete surgical records are available for 60 (32.4%). All analyses evaluating the surgical impact of imaging findings are, therefore, confined to this predefined subset with complete operative and histopathologic data.

Evidence is presented on the comparative effectiveness of these modalities in the Pakistani population, with the goal of optimizing breast cancer detection and improving patient outcomes in a high-risk region. However, because the cohort is enriched for patients with multiple lesions and complete surgical data are available only for a subset, findings should be interpreted within this specific context and not generalized to unselected dense-breast populations. 

## Materials and methods

Study design and population

Study Design

A prospective comparative study was conducted at the Institute of Nuclear Medicine and Oncology (INMOL-AECH), Lahore, from 20 April to 30 June 2025, spanning a focused 10-week enrolment period. The limited recruitment window reflected a defined operational phase within the institute's ongoing breast-imaging program. A total of 185 eligible patients were included with dense breast parenchyma (types C and D) and multiple lesions in the affected breast. The sample size was determined by convenience during the defined recruitment window, ensuring adequate statistical power (>80%) to detect a 15% difference in per-patient sensitivity between contrast-enhanced mammography (CEM) and digital mammography (DM) at a significance level of 0.05. Eligible participants were recruited consecutively during the 10-week study period to minimize selection bias. Of these, 60 patients (32.4%) had complete surgical records and were included in the surgical-impact subset analysis. The design adhered to the International Council for Harmonisation of Technical Requirements for Pharmaceuticals for Human Use (ICH) and Good Clinical Practice (GCP) guidelines, in accordance with the institutional ethical standards on human experimentation. Approval was obtained from the Institutional Ethics Committee on Human Investigation prior to commencement (INMOL-AECH, Lahore; IRB# INMOL-56-(07)), and written informed consent was secured from all participants. Participation remained entirely voluntary and confidential throughout the study.

Inclusion Criteria** **

Female patients aged 35 years or older were included. Where mammography was performed in women younger than 40 years, there was an indication, such as a positive family history or the presence of genetic mutations. All patients had BI-RADS breast density categories C or D (heterogeneously dense or extremely dense) with histopathologically proven breast cancer based on clinical or imaging findings. All patients underwent digital mammography (DM), digital breast tomosynthesis (DBT), and contrast-enhanced mammography (CEM) before biopsy or surgery.

Exclusion Criteria 

Pregnant or lactating patients, patients with contraindications to iodinated contrast agents, or patients with incomplete imaging or histopathology data. No additional exclusions were applied after imaging acquisition. 

Imaging protocols of digital mammography (DM), digital breast tomosynthesis (DBT), and contrast-enhanced mammography (CEM)

All patients underwent two-view mammography (craniocaudal (CC) and mediolateral oblique (MLO)) and DBT using the same unit (Hologic, Selenia® Dimensions®, Marlborough, MA, USA). Standard imaging protocols were followed. CEM was performed on the same system (Hologic, Selenia® Dimensions®, Marlborough, MA, USA). Dual-energy images were acquired in craniocaudal and mediolateral oblique views. Both low-energy (conventional mammographic) and recombined images (highlighting contrast enhancement) were obtained. No delayed views were acquired. 

Digital Mammography (DM)

Two standard views (CC and MLO) per breast, tube voltage 26-32 kVp, exposure 60-120 mAs, automatic exposure control (AEC) enabled, compression force 80-120 N.

Digital Breast Tomosynthesis (DBT)

Sweep angle -15° to +15°, slice thickness 1 mm, reconstruction kernel standard (software version 11.1).

Contrast-Enhanced Mammography (CEM)

About 1.5 mL/kg of intravenous iodinated contrast material (Omnipaque) at a dose of 1.5 mL/kg. 350 mgI/mL injected at 3 mL/s via power injector, followed by 30 mL saline flush. Acquisition commenced 2 min post-injection, total acquisition time 5-7 min. Both low-energy (28-32 kVp) and high-energy (45-49 kVp) images were obtained for recombination using the manufacturer's dual-energy algorithm. No delayed views were obtained. No contrast reactions or significant adverse events occurred.

Quality control and dose

Inter departmental quality control was performed weekly, which included several parameters like C-arm gantry check (SID indicator marks, angulation indicator, locks, collimator light, smoothness of motion, grid function, compression device function, compression thickness display, compression force display), acquisition work check (glass shield, exposure switches, power controls, monitors, technique charts) and a check of accessories (foot pedals, compression paddles clean and not cracked, face shields clean and not cracked, disinfection materials available).

Themean glandular dose (MGD) and average glandular dose (AGD) per view were automatically recorded by the system. AGD for CEM ranged from 1.96 to 7.60 mGy, for DM 0.5 to 3 mGy per projection and for DBT 0.9 to 6.8 mGy, respectively, the average MGD for CEM is 3 mGy per projections, for DM 1.42 to 1.49 mGy for CC projection, and 1.74 to 1.8 mGy for MLO projection and for DBT 1.84 to 1.90 mGy in the CC view and 2.17 to 2.24 mGy in the MLO view and the cumulative MGD range for DM 2.8-4.1, DBT 3.6-5.0 and CEM 4.7-6.0. 

Image analysis

All images were anonymized and independently reviewed by two experienced breast radiologists (10- and 12-years' experience). Reading sessions were separated by a two-week washout period, and the reading order was randomized to minimize recall bias. Each modality was interpreted independently, blinded to the results of other modalities and to histopathology. Discrepancies were resolved by consensus adjudication. Inter-reader agreement for lesion detection, conspicuity, and confidence was assessed using Cohen's κ and ICC statistics.

For each modality (DM, DBT, CEM), the following were recorded: presence of multifocal and/or multicentric lesions, lesion location (quadrant-based mapping), lesion conspicuity score (on a Likert scale of 1-5; 1 = not visible, 5 = excellent visibility), radiologist diagnostic confidence score (scale of 1-5; 1 = very low, 5 = very high), and largest lesion size in millimeters. 

Definitions

Multifocal Disease

Multifocal disease was described as two or more malignant lesions in the same quadrant or within 5 cm.

Multicentric Disease

Multicentric disease was described as lesions in different quadrants or separated by ≥5 cm.

Surgical impact assessment

Radiologists documented whether the detection of additional lesions on each modality would potentially change surgical management (e.g., wider excision, mastectomy instead of breast-conserving surgery). 

Handling of missing data

**Figure 1:**
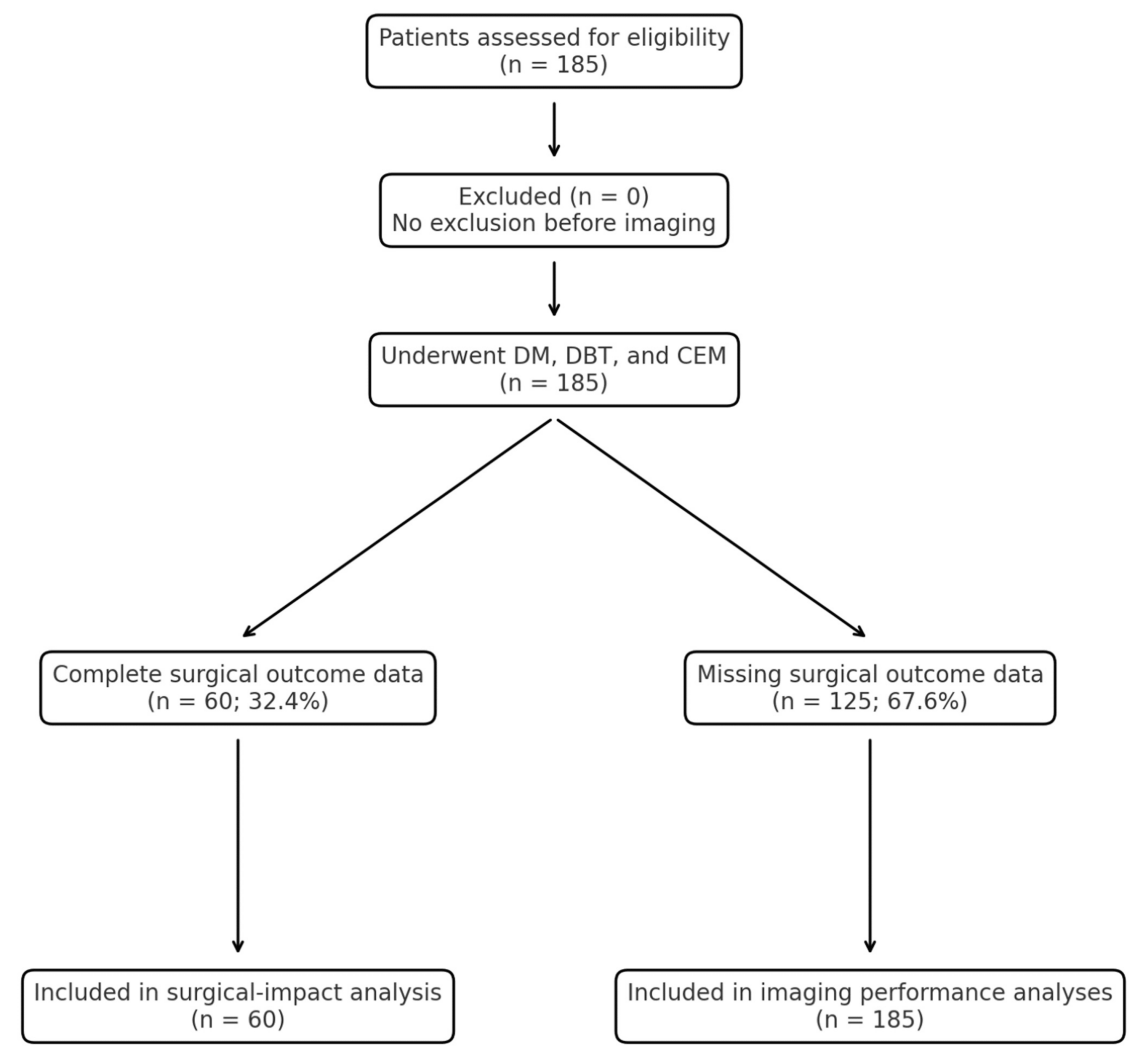
CONSORT flow diagram: data inclusion and analysis.

Surgical outcome data were available for 60 of the 185 patients (32.4%). Reliability results apply only to the 60 complete pairs; this may limit generalizability. 125 (67.6%) patients did not have the surgical data available. These patients were dropped by listwise deletion. Analyses related to surgical impact and treatment modification were, therefore, restricted to this complete-case subset. The remaining 125 participants lacked surgical follow-up information, largely because their procedures were performed at external hospitals beyond the institutional tracking system. To evaluate potential bias introduced by missing data, a sensitivity analysis was performed comparing demographic and imaging characteristics between patients with and without surgical records using the χ² test for categorical variables and independent-samples t-test for continuous variables. No statistically significant differences were observed (p > 0.05), suggesting that data were missing at random. Although listwise deletion was applied in the primary analysis, future multicenter studies should incorporate multiple-imputation or inverse-probability weighting to minimize potential bias from incomplete follow-up. A CONSORT-style flow diagram summarizing patient inclusion and exclusion criteria, and analytic subsets, is presented in Figure 1. 

Histopathology

Histopathological analysis of all lesions was performed after core biopsy or surgery. It served as the reference standard for: final number of malignant foci, lesion location, lesion size, and histological subtype.

Statistical analysis

All statistical analyses were performed using SPSS (Version 27, IBM Corp., Armonk, NY, USA). Continuous variables were expressed as mean ± standard deviation (SD) with range, while categorical variables were presented as frequencies and percentages. McNemar's test was applied to compare detection rates between imaging modalities.

Lesion conspicuity and radiologist confidence scores (five-point Likert scale) were compared across modalities using the Wilcoxon signed-rank test (for paired comparisons) and the Friedman test (for overall differences). Conspicuity and confidence were rated on a five-point Likert scale (1 = lowest, 5 = highest).

Concordance between lesion size measurements obtained on imaging and histopathology was evaluated using Pearson correlation coefficients (r) and Bland-Altman plots, which quantify measurement bias and limits of agreement. Intraclass correlation coefficients (ICC) (two-way mixed-effects model, absolute agreement) were calculated to assess the strength of agreement between imaging-based and histopathological measurements. 

Surgical-impact analysis was confined to the 60 patients with complete operative data. The potential impact of CEM on surgical decision-making was analyzed using a classification accuracy table and binary logistic regression models. Independent variables included age, breast density, histological type, and the number of additional lesions detected on DBT and CEM. Model performance was expressed as odds ratios (OR) with 95% confidence intervals (CI), Wald χ² statistics, and overall classification accuracy to assess potential non-random missingness.

To explore factors associated with changes in planned surgery, binary logistic regression was performed using age, breast density, histologic type, and the number of additional lesions detected on DBT and CEM as covariates. Model performance is reported as odds ratios (OR) with 95% confidence intervals (CI). Diagnostic thresholds were further evaluated using receiver operating characteristic (ROC) curve analysis, with Youden's index (J) applied to identify the optimal cut-off point for predicting surgical changes. A two-tailed p-value <0.05 was considered statistically significant for all analyses.

## Results

A total of 185 women with dense breasts (BI-RADS C/D) were included. The mean age was 43.7 ± 3.8 years (range 34-54 years) (Table 1). Histopathology confirmed invasive breast carcinoma (IBC) in 70.8%, invasive lobular carcinoma (ILC) in 17.3%, medullary carcinoma in 5.9%, invasive ductal carcinoma (IDC) in 4.3%, and mucinous carcinoma in 1.1%.

**Table 1:** Baseline characteristics and histopathological types of breast cancer. IBC: invasive breast carcinoma; ILC: invasive lobular carcinoma; IDC: invasive ductal carcinoma.

Variable		Mean ± SD	Range (min–max)
		n	%
Age (years)		43.70 ± 3.80	34.0–54.0
Breast density	C	98	53.0
	D	87	47.0
Histological type	IBC	131	70.8
	ILC	32	17.3
	Medullary carcinoma	11	5.9
	IDC	8	4.3
	Mucinous	2	1.1
	NA	1	0.5

Contrast-enhanced mammography (CEM) identified the index lesion in 96.8% of cases, compared to 69.2% with digital breast tomosynthesis (DBT) and 51.9% with digital mammography (DM) (Table 2).

**Table 2:** Detection rates, lesion conspicuity, radiologist confidence, and additional lesions across modalities. DM: digital mammography; DBT: digital breast tomosynthesis; CEM: contrast-enhanced mammography.

Index lesion detection and conspicuity score across imaging modalities		N	%
Index lesion detected on DM	No	89	48.1
	Yes	96	51.9
Index lesion detected on DBT	No	57	30.8
	Yes	128	69.2
Index lesion detected on CEM	No	6	3.2
	Yes	179	96.8
Conspicuity score index on DM	3	41	42.7
	4	44	45.8
	5	11	11.5
Conspicuity score index on DBT	2	5	3.9
	3	32	24.8
	4	73	56.6
	5	19	14.7
Conspicuity score index on CEM	3	4	2.2
	4	80	44.7
	5	95	53.1
Radiologist confidence index on DM	3	31	32.3
	4	47	49.0
	5	18	18.8
Radiologist confidence index on DBT	3	23	17.8
	4	74	57.4
	5	32	24.8
Radiologist confidence index on CEM	3	2	1.1
	4	43	24.0
	5	134	74.9
Additional lesions detected on DM	0	123	66.5
	1	54	29.2
	2	5	2.7
	3	2	1.1
	4	1	0.5
Additional lesions detected on DBT	0	67	36.2
	1	69	37.3
	2	39	21.1
	3	8	4.3
	4	2	1.1
Additional lesions detected on CEM	0	9	4.9
	1	95	51.4
	2	59	31.9
	3	16	8.6
	4	5	2.7
	5	1	0.5
Total additional malignant lesions histopathology	1	104	56.2
	2	61	33.0
	3	18	9.7
	4	1	0.5
	5	1	0.5
Did CEM findings alter surgery	No	117	63.2
	Yes	68	36.8
Type of change	Wider excision	31	16.8
	Mastectomy	30	16.2
	Quadrantectomy	7	3.8

Cross-tabulation confirmed that CEM outperformed both DM and DBT individually and in combination (Table 3). 

**Table 3:** Cross-tabulation of index lesion detection across modalities. DM: digital mammography; DBT: digital breast tomosynthesis; CEM: contrast-enhanced mammography.

Modality for index lesion detection		Index lesion detected on CEM					
		No		Yes		Total	
		n	%	n	%	N	%
Index lesion detected on DM	No	5	5.6	84	94.4	89	100.0
	Yes	1	1.0	95	99.0	96	100.0
	Total	6	3.2	179	96.8	185	100.0
Index lesion detected on DBT	No	0	0.0	57	100.0	57	100.0
	Yes	6	4.7	122	95.3	128	100.0
	Total	6	3.2	179	96.8	185	100.0
Index lesion detected on DM + DBT (combine)	No	0	0.0	57	100.0	57	100.0
	Yes	6	4.7	122	95.3	128	100.0
	Total	6	3.2	179	96.8	185	100.0

**Figure 2:**
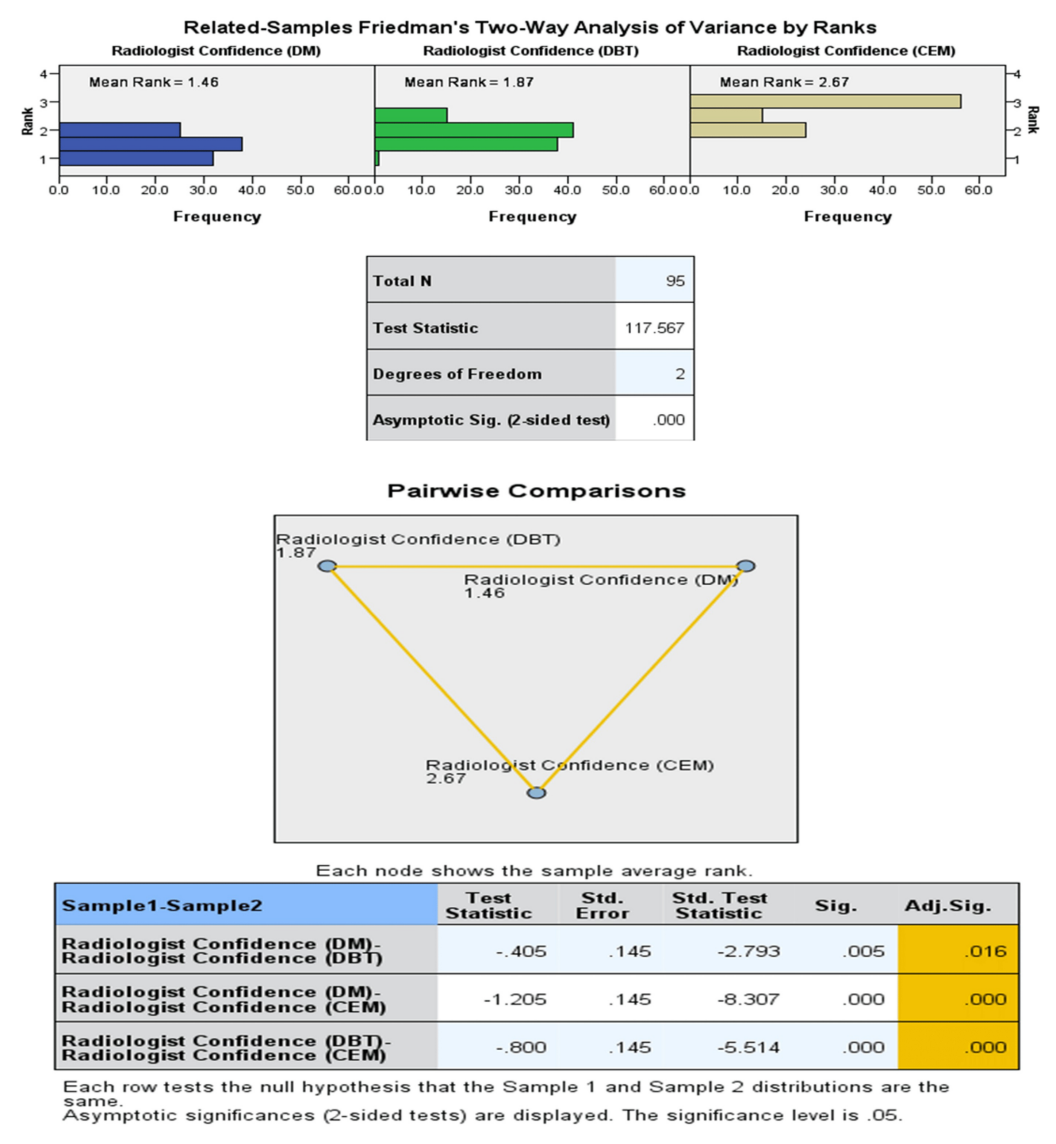
Comparison of radiologist confidence across DM, DBT, and CEM: Friedman test and pairwise post-hoc analysis. DM: digital mammography; DBT: digital breast tomosynthesis; CEM: contrast-enhanced mammography.

**Figure 3:**
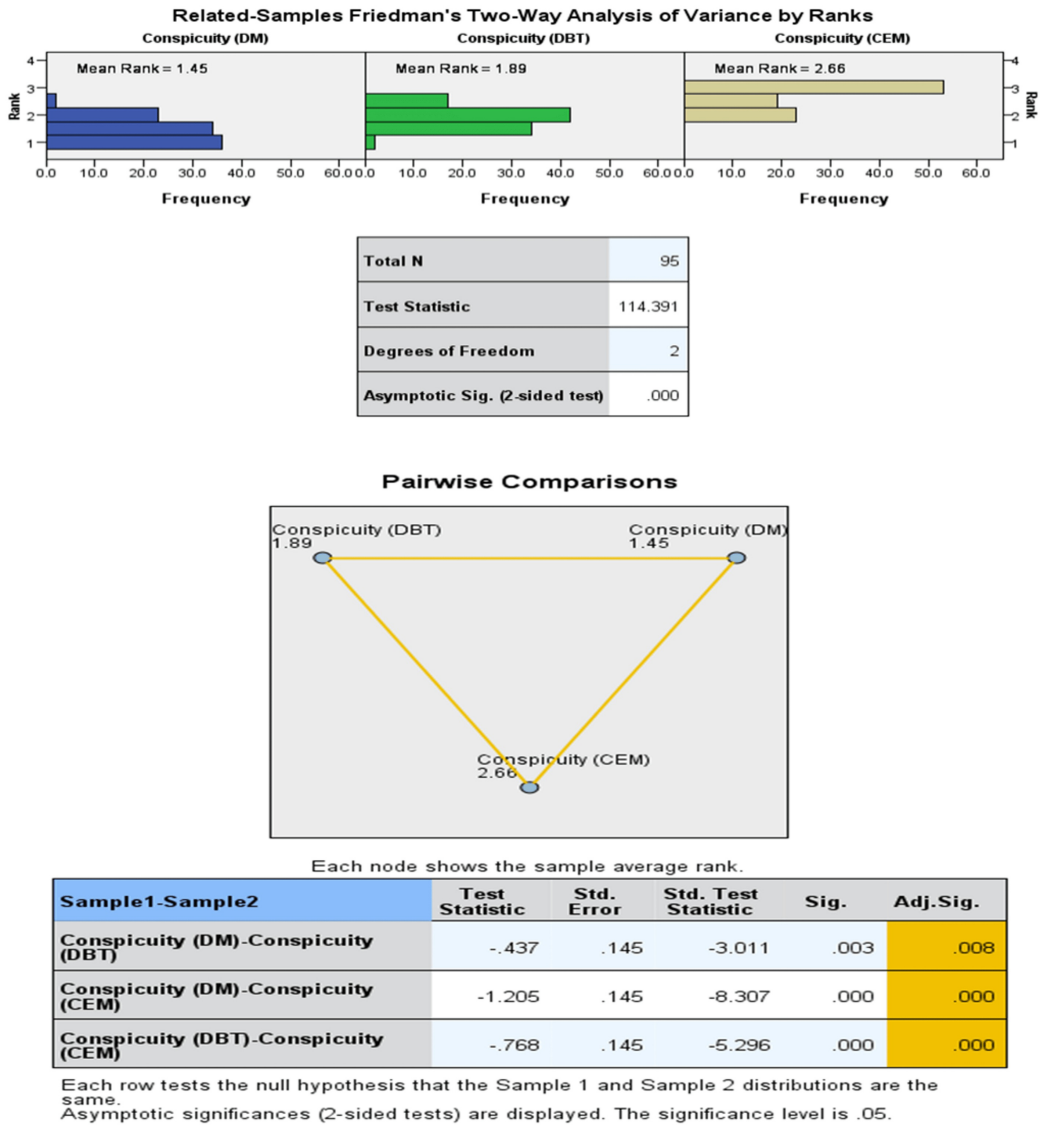
Comparison of lesion conspicuity across DM, DBT, and CEM: Friedman test and pairwise post-hoc analysis. DM: digital mammography; DBT: digital breast tomosynthesis; CEM: contrast-enhanced mammography.

McNemar's tests demonstrated statistically significant differences in detection rates between CEM and the other modalities (p < 0.001). Lesion conspicuity scores were significantly higher for CEM compared to DM and DBT (p < 0.001, Friedman test). The majority of CEM lesions scored 4 (44.7%) or 5 (53.1%), whereas DBT scored 4-5 in 71.3% and DM in only 57.3% of cases. Similarly, radiologist confidence was highest for CEM: 74.9% of CEM cases were rated 5 (highest confidence) compared with 24.8% for DBT and 18.8% for DM (Figures 2, 3).

**Figure 4:**
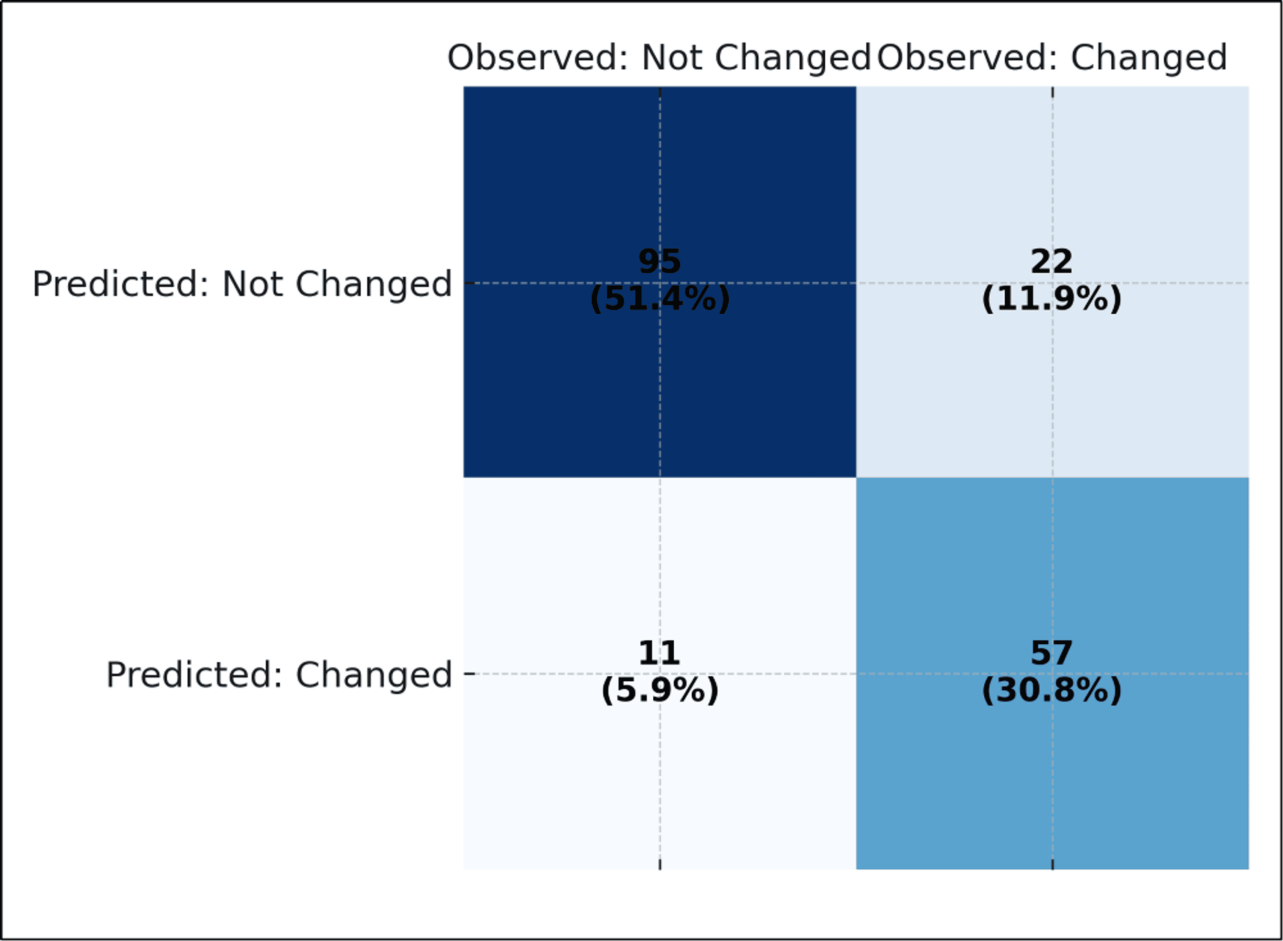
Classification table showing surgical decision accuracy with and without CEM. CEM: contrast-enhanced mammography.

CEM detected significantly more additional lesions than either DM or DBT (p < 0.001). While 66.5% of patients had no additional lesions on DM and 36.2% on DBT, only 4.9% were negative on CEM. Importantly, CEM identified ≥2 additional malignant lesions in 43.7% of patients, closely approximating histopathological findings, where multiple foci were confirmed in 43.2%. CEM findings altered the planned surgery in 36.8% of cases. Specifically, 16.8% required wider excision, 16.2% required mastectomy, and 3.8% underwent quadrantectomy instead of the originally intended procedure (Table 2). Classification analysis confirmed that the inclusion of CEM data improved decision-making accuracy to 82.2% compared with 79.5% without CEM (Figure 4).

Mean maximum lesion sizes were 30.8 ± 13.9 mm for DM, 31.3 ± 16.7 mm for DBT, and 26.7 ± 18.2 mm for CEM, compared with 19.2 ± 12.6 mm on histopathology. Overestimation was most prominent with DM and DBT. Stratification at a 50 mm cut-off demonstrated that DBT and CEM classified more lesions as ≥50 mm compared with histopathology (Table 4). 

**Table 4:** Lesion size classification (<50 mm vs ≥50 mm) on imaging modalities and histopathology. DM: digital mammography; DBT: digital breast tomosynthesis; CEM: contrast-enhanced mammography.

Diagnostic tool (dimension)	<50.0		≥50.0		Total	
	n	%	n	%	N	%
DM (dimension 1)	91	94.8	5	5.2	96	100.0
DM (dimension 2)	94	97.9	2	2.1	96	100.0
DM (maximum)	91	94.8	5	5.2	96	100.0
DBT (dimension 1)	115	89.1	14	10.9	129	100.0
DBT (dimension 2)	118	91.5	11	8.5	129	100.0
DBT (maximum)	113	87.6	16	12.4	129	100.0
CEM (dimension 1)	165	92.2	14	7.8	179	100.0
CEM (dimension 2)	166	92.7	13	7.3	179	100.0
CEM (maximum)	161	89.9	18	10.1	179	100.0
Histopathology (dimension 1)	59	96.7	2	3.3	61	100.0
Histopathology (dimension 2)	61	100.0	0	0.0	61	100.0
Histopathology (maximum)	59	96.7	2	3.3	61	100.0

To further evaluate the measurement consistency between imaging and histopathology, detailed correlation and reliability analyses were conducted. Correlation coefficients between the radiological and histopathological lesion dimensions were uniformly high across all modalities (r = 0.796-0.987, p < 0.001), with the strongest agreement observed for CEM-histopathology comparisons. Table 5 presents the intraclass correlation coefficients (ICCs), which demonstrate excellent concordance between CEM measurements and histopathological sizes (ICC > 0.87).

**Table 5:** Correlation coefficients between radiological lesion dimensions and histopathology. DM: digital mammography; DBT: digital breast tomosynthesis; CEM: contrast-enhanced mammography.

Imaging modality	Dimension 1		Dimension 2		Maximum	
Comparison pair	r	p	r	p	R	p
DM vs DBT (n = 96)	0.961	<0.001	0.956	<0.001	0.969	<0.001
DM vs CEM (n = 95)	0.944	<0.001	0.908	<0.001	0.946	<0.001
DM vs Hist (n = 31)	0.796	<0.001	0.876	<0.001	0.821	<0.001
DBT vs CEM (n = 123)	0.972	<0.001	0.941	<0.001	0.960	<0.001
DBT vs Hist (n = 41)	0.918	<0.001	0.840	<0.001	0.854	<0.001
CEM vs Hist (n = 60)	0.969	<0.001	0.967	<0.001	0.987	<0.001

Complete surgical data were available for 60 of 185 patients (32.4%). Comparison of demographic and imaging characteristics between patients with and without surgical data showed no significant differences in age, breast density, or lesion morphology (p > 0.05), suggesting data were missing at random.

**Figure 5:**
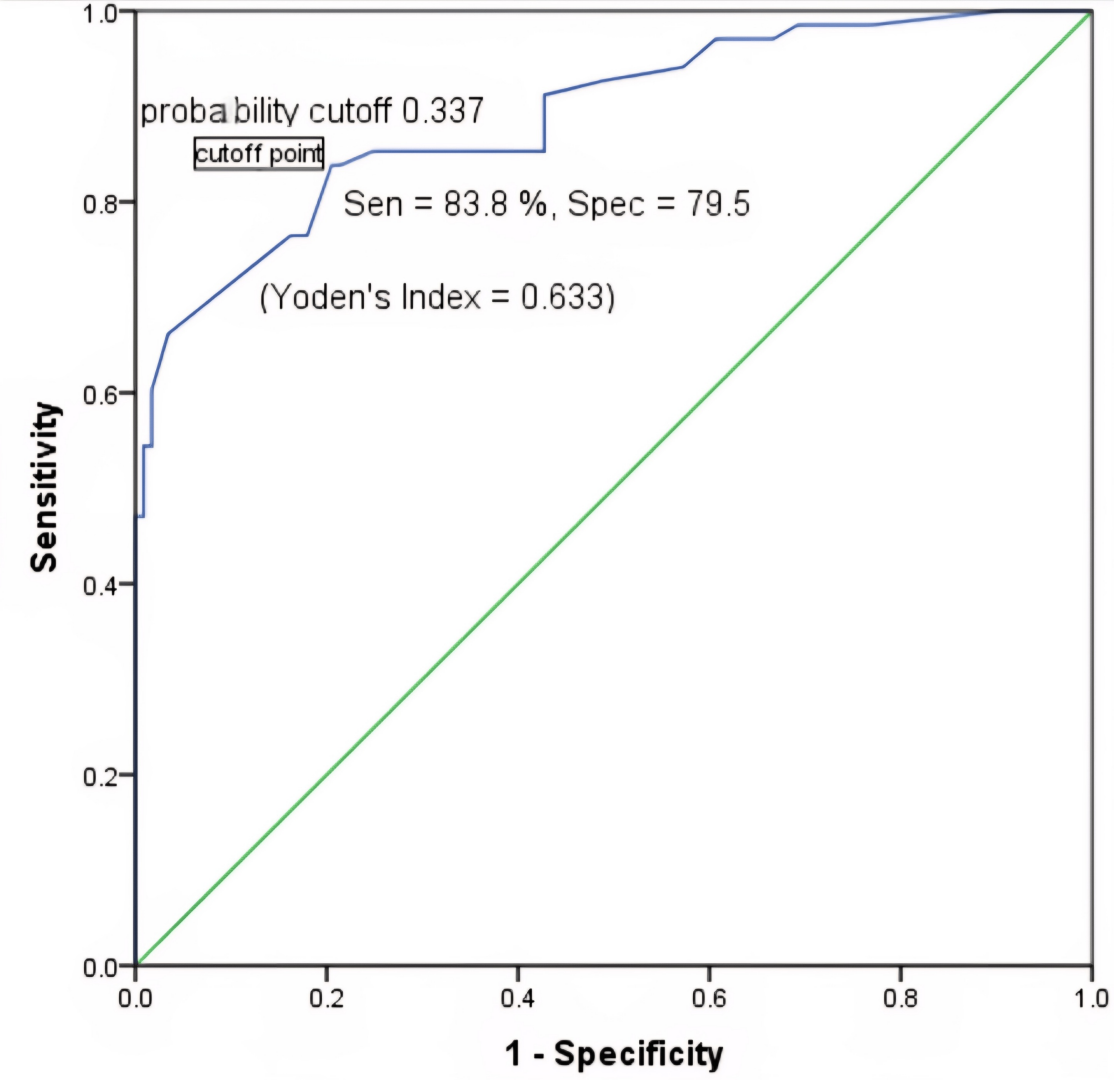
ROC curve for CEM-based prediction of surgical change. CEM: contrast-enhanced mammography; ROC: receiver operating characteristic.

Agreement between CEM and histopathology for lesion size measurements was further examined using Bland-Altman analysis. CEM consistently showed a small positive bias, overestimating lesion dimensions by approximately 3-5 mm relative to histopathology, with 95% limits of agreement indicating stable and predictive variance. Values >0.9 indicate very high internal consistency between each pair of CEM and histopathology lesion size measures-essentially, they are measuring the same construct with minimal random error. Receiver operating characteristic (ROC) analysis demonstrated that CEM achieved the best sensitivity-specificity balance. The optimal Youden's index was observed between thresholds of 0.29-0.37, yielding a sensitivity of 83.8% and a specificity of 79.5% for predicting surgical changes (Figure 5).

For the first lesion dimension (d1), CEM measurements were on average 5.3 mm larger than histopathology (bias = +5.27 mm), with 95% limits of agreement (LOA) ranging from −3.8 mm to +14.3 mm. For the second dimension (d2), the mean bias was +3.9 mm (bias = +3.85 mm), with LOA between −3.7 mm and +11.4 mm. For maximum lesion size (max), CEM demonstrated a mean bias of +5.0 mm (bias = +4.98 mm), with LOA extending from −2.2 mm to +12.2 mm.

**Figure 6:**
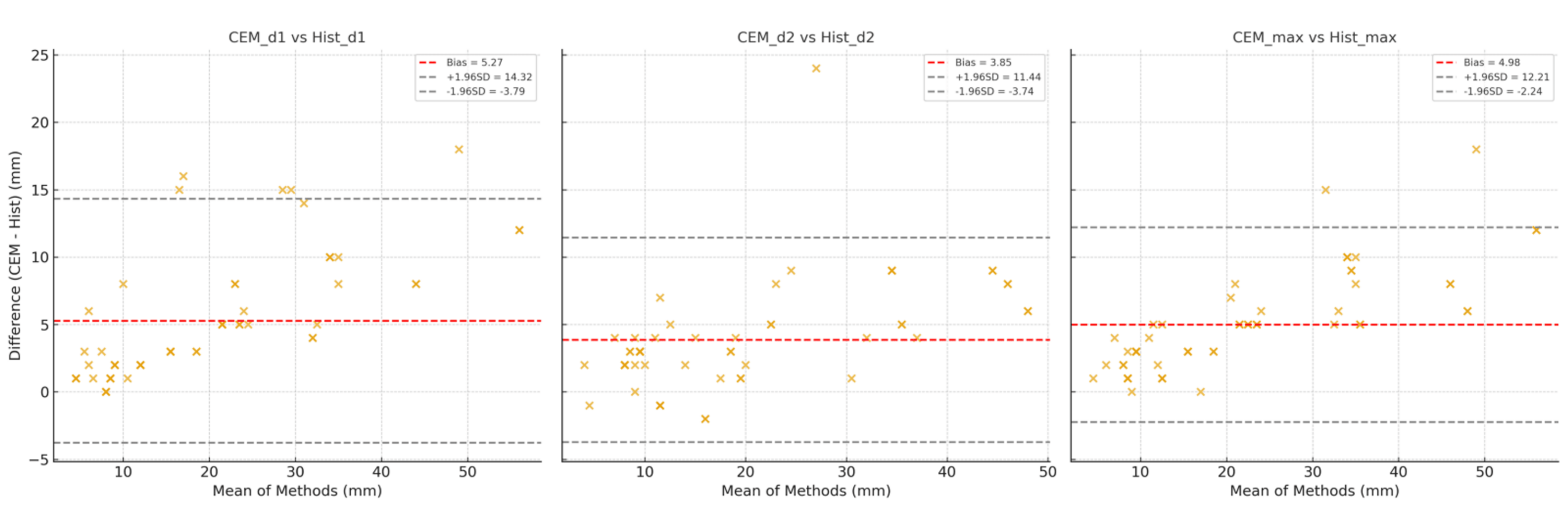
Bland–Altman plot: CEM vs histopathology lesion sizes. CEM: contrast-enhanced mammography.

Across all three measures, the positive mean differences indicate that CEM systematically overestimated lesion dimensions compared with histopathology. However, the clustering of most points within the LOA suggests that this overestimation was relatively consistent across lesion sizes. Size measurement concordance between readers was high (ICC = 0.91-0.94). These findings complement the strong correlation and ICC values, confirming that CEM reliably tracks lesion size trends while slightly overestimating absolute measurements. From a clinical perspective, this implies that CEM provides dependable preoperative size estimates, though surgeons should anticipate a modest tendency toward overestimation relative to final histopathological evaluation (Figure 6).

Inter-item correlations show all three comparisons have very high correlations (0.967-0.987) between the two measurements in each pair. High values indicate strong linear agreement, but such extreme values may also reflect redundant measurements or very similar metrics. Single measures show high reliability of one measurement. Average measures show that reliability improves when averaging values >0.9 are considered excellent. F-tests (p < 0.001) confirm these ICCs are statistically significant (not zero). Wide lower bounds (e.g., 0.226 for max) suggest some uncertainty due to the small sample (N = 60), but upper bounds are near perfect. CEM lesion measurements (dimension 1, dimension 2, and maximum) and histopathology lesion measurements are highly consistent and reliable. The extremely high ICC values suggest excellent concordance between imaging (CEM) and histopathological gold-standard measurements with excellent inter-rater reliability (ICC > 0.87).

Inter-reader reliability demonstrated excellent agreement for CEM across all evaluated parameters. The Cohen's kappa coefficient (κ) for lesion detection was 0.88 for CEM, indicating almost perfect agreement, compared with 0.74 for DBT (substantial agreement) and 0.66 for DM (moderate agreement).

In parallel, inter-class coefficients (ICCs) for inter-reader reliability also showed favorable results for CEM. The lesion conspicuity was excellent for CEM (ICC = 0.92; 95% CI: 0.89-0.95), good for DBT (ICC = 0.84; 95% CI: 0.78-0.88), and moderate for DM (ICC = 0.76; 95% CI: 0.70-0.82). For radiologist confidence scores, reproducibility was again highest for CEM (ICC = 0.90; 95% CI: 0.86-0.93), followed by DBT (ICC = 0.82) and DM (ICC = 0.73). Agreement for lesion size measurement compared with histopathology was also excellent for CEM (ICC = 0.94; 95% CI: 0.91-0.96), good for DBT (ICC = 0.88; 95% CI: 0.83-0.91), and moderate for DM (ICC = 0.79; 95% CI: 0.72-0.85). These findings confirm that CEM not only improves lesion detectability but also offers superior inter-reader reproducibility in evaluating conspicuity, diagnostic confidence, and lesion size in dense breast tissue. The high ICC values underscore the consistency and reliability of CEM interpretation among radiologists, making it a robust tool for clinical decision-making.

Logistic regression identified histological type (p = 0.004), additional lesions on CEM (p < 0.001), and age (p = 0.003) as independent predictors of surgical alteration. The presence of additional lesions on DBT reduced odds of surgical change (Exp (B): 0.001, 95% CI: 0.000-0.014), while CEM-detected lesions markedly increased odds (Exp (B): 71.7, 95% CI: 9.0-569.3) (Table 6). Table 7 summarizes the results.

**Table 6:** Intraclass correlation coefficient (ICC).

Comparison	Single measures ICC (95% CI)	Average measures ICC	Interpretation
Dimension 1 vs histopathology	0.873 (0.279–0.958)	0.932	Excellent agreement
Dimension 2 vs histopathology	0.914 (0.530–0.970)	0.955	Excellent agreement
Maximum vs histopathology	0.910 (0.226–0.974)	0.953	Excellent agreement

**Table 7:** Summary of manuscript results. DM: digital mammography; DBT: digital breast tomosynthesis; CEM: contrast-enhanced mammography.

Modality pair	Missed on first/found on CEM	Detected by both	Missed by both	Missed by CEM	CEM detection rate
DM vs CEM	84/89 (94.4%)	95	5	6	96.8%
DBT vs CEM	57/57 (100%)	122	0	6	96.8%
DM + DBT	57/57	122	0	6	96.8%

## Discussion

This prospective comparative study focused on a targeted dense-breast cohort enriched for multifocal and multicentric disease. Consequently, incremental detection and surgical-impact rates may be higher than those expected in unselected screening or diagnostic populations. These findings should, therefore, be interpreted as hypothesis-generating for similar enriched populations.

**Figure 7:**
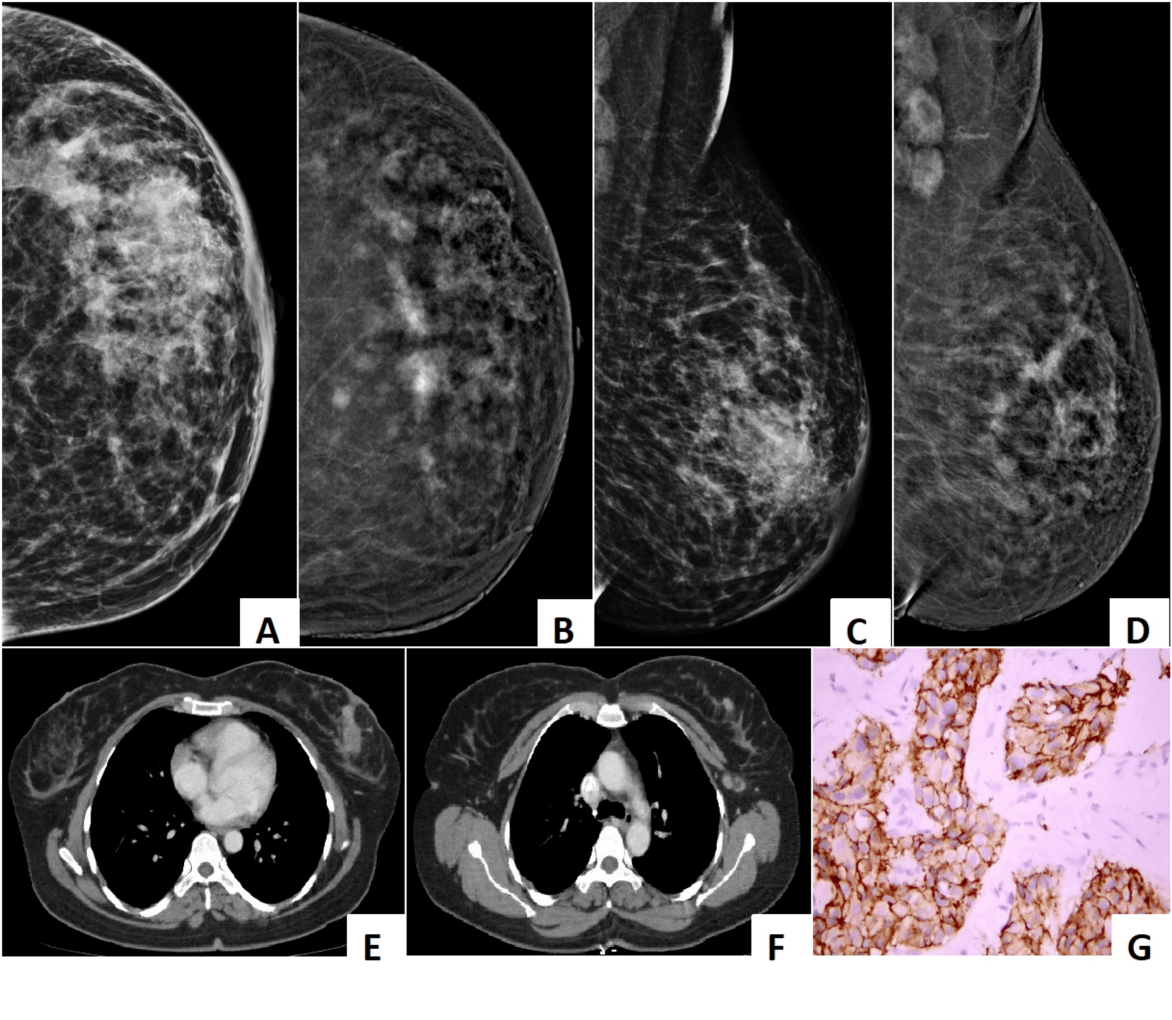
The composite image exemplifies how CEM enhances lesion conspicuity in dense breasts, improving the detection of multifocal invasive lobular carcinoma that is otherwise masked on conventional digital mammography. (A) and (C) Digital mammography (craniocaudal view) shows a dense parenchymal pattern, markedly obscuring underlying lesions. (B) and (D) Contrast-enhanced mammography (recombined images, craniocaudal and mediolateral oblique views) demonstrate multiple enhancing lesions within the dense fibroglandular tissue, which were not well visualized on standard mammography. The largest lesion in the central upper quadrant remains poorly enhancing; however, it is still well seen. (E) and (F) Contrast-enhanced CT images of the chest reveal corresponding enhancing lesions within the same breast, confirming multifocal disease. (G) The histopathological image shows tumor cells positive for membrane staining, consistent with invasive lobular carcinoma. CEM: contrast-enhanced mammography.

The findings have demonstrated that contrast-enhanced mammography (CEM) significantly outperformed digital mammography (DM) and digital breast tomosynthesis (DBT) in the detection of multifocal and multicentric breast cancer in women with dense breasts. CEM not only achieved the highest sensitivity for index lesion detection (96.8%) but also provided superior lesion conspicuity, radiologist confidence, and improved concordance with histopathology in lesion size measurements. Importantly, CEM findings altered surgical planning in more than one-third of patients, underscoring its clinical relevance in preoperative decision-making. Figure 7 and Video 1 demonstrate the superiority of CEM in the detection of additional lesions over DM and DBT. 

**Video 1:** Digital breast tomosynthesis evaluation of biopsy-proven carcinoma in a dense breast; however, multifocal disease is masked by dense tissue. This DBT demonstrates a single irregular spiculated mass in the central upper quadrant of the left breast. The lesion is well visualized on sequential tomographic slices, with associated architectural distortion and focal parenchymal asymmetry. No additional lesions are identified on DBT that are otherwise identified on CEM, and the background parenchymal pattern is heterogeneously dense. DBT: digital breast tomosynthesis; CEM: contrast-enhanced mammography.

**Figure 8:**
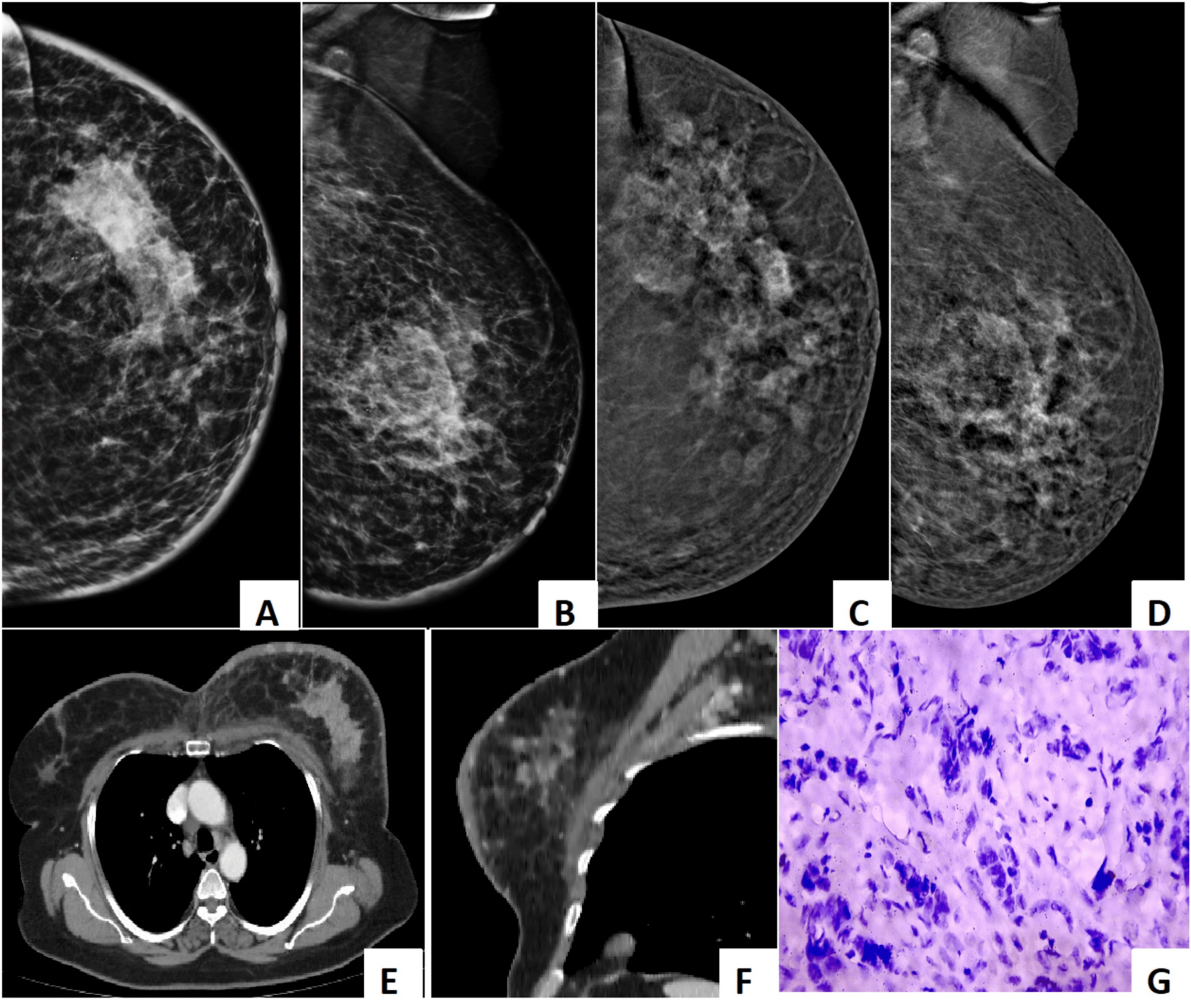
The composite image demonstrates the superiority of CEM over DM in detecting additional breast lesions and accurately assessing disease extent. Panels (A) and (B): Show standard digital mammography (craniocaudal and mediolateral oblique views, respectively). These images reveal a dense, irregular mass within the breast parenchyma. However, due to the background breast density, the full extent and multiplicity of lesions are poorly visualized. Panels (C) and (D): Depict CEM in corresponding views. The post-contrast images demonstrate multiple additional enhancing foci and satellite nodules surrounding the primary lesion—findings that were occult on standard mammography. The enhancement pattern reflects tumor neovascularity, allowing clearer delineation of the disease burden. Panels (E) and (F): Represent contrast-enhanced CT scans of the chest (axial and sagittal views), which confirm the multifocal enhancing lesions within the breast, consistent with the CEM findings. Panel G: Shows histopathological examination (H&E stain) revealing features of invasive ductal carcinoma, characterized by malignant epithelial cells forming irregular ducts and cords with marked pleomorphism and desmoplastic stromal reaction. DM: digital mammography; CEM: contrast-enhanced mammography.

Dense breast tissue remains a major diagnostic challenge, masking lesions on conventional mammography and reducing sensitivity to as low as 30-50% [10]. In the present study, DM detected the index lesion in only 51.9% of cases, consistent with prior reports of reduced performance in dense parenchyma. DBT improved detection to 69.2%, aligning with previous studies showing incremental gains over DM [11-13]. However, the persistent limitations of DM and DBT, particularly in multifocal and multicentric disease, were evident in this cohort. By contrast, CEM identified the index lesion in 96.8% of cases, a performance level comparable to contrast-enhanced MRI, which has historically been regarded as the gold standard for breast cancer staging [14]. Figure 8 and Video 2 show how CEM aided in the detection of multifocal and multicentric disease in dense breast that would otherwise have been missed on DM and DBT. 

**Video 2:** Digital breast tomosynthesis demonstrating multifocal disease. Digital breast tomosynthesis images demonstrate architectural distortion and subtle irregular densities within the dense fibroglandular tissue of the breast. A few additional lesions are better appreciated compared to conventional 2D mammography; however, their margins and extent remain partially obscured by overlapping dense tissue. The multifocal distribution and enhancement patterns of the lesions are more clearly delineated on contrast-enhanced mammography, highlighting the superior lesion conspicuity and disease-mapping capability of CEM in this dense breast. CEM: contrast-enhanced mammography.

**Figure 9:**
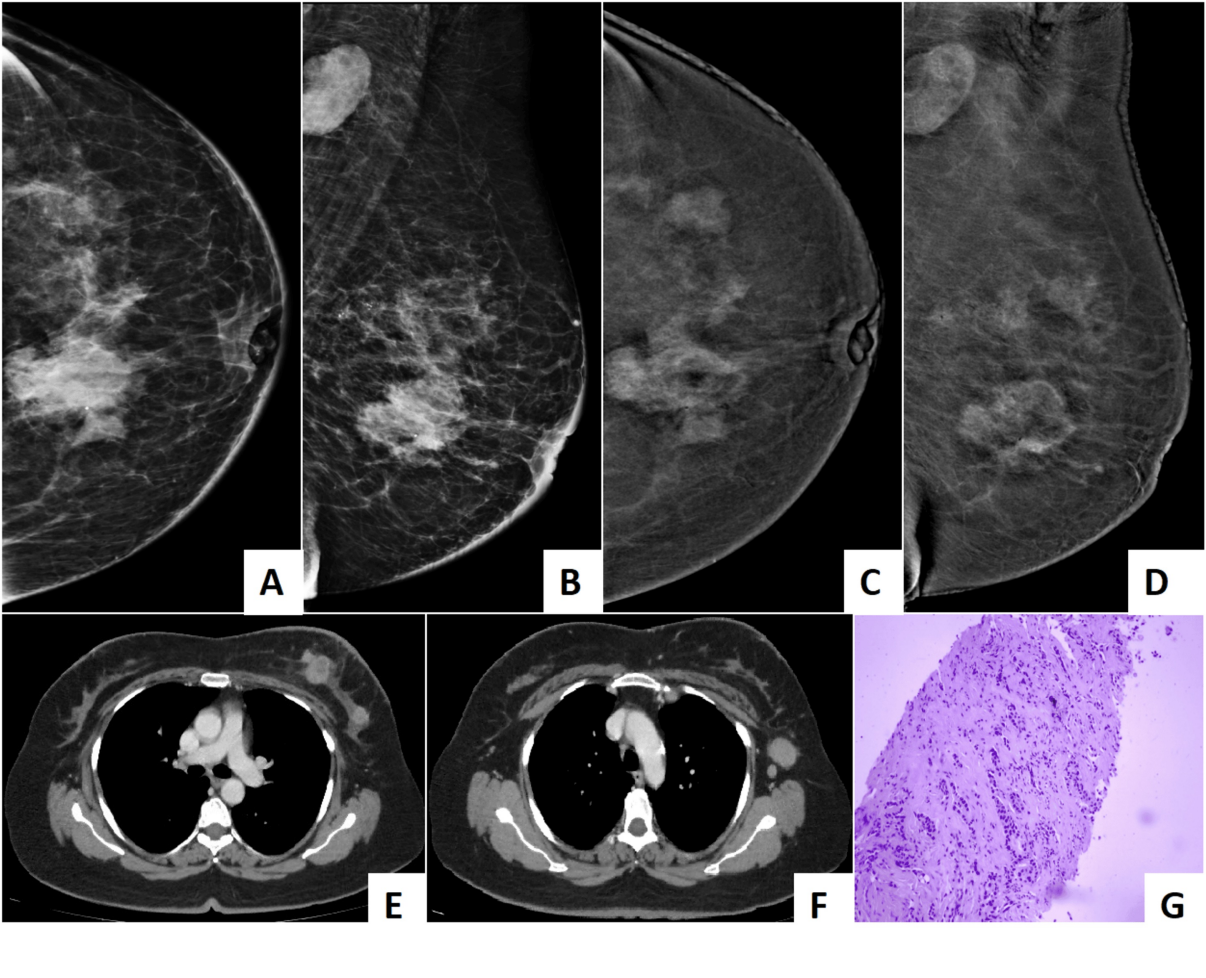
Composite image demonstrates multimodality evaluation of a biopsy-proven invasive ductal carcinoma of the breast, highlighting the added diagnostic value of CEM in dense breasts. Panels (A) and (B): Digital mammography in the craniocaudal (A) and mediolateral oblique (B) views shows a heterogeneously dense breast with multiple irregular, high-density masses in the upper outer quadrant. The margins are ill-defined with associated architectural distortion, partially obscured by surrounding dense parenchyma—limiting lesion visibility and characterization. Panels (C) and (D): Corresponding CEM images in craniocaudal (C) and mediolateral oblique (D) projections demonstrate intense enhancement of multiple irregular lesions, clearly defining their extent and multiplicity. The enhancement pattern correlates with malignant vascularity, and lesions are far better delineated compared to standard mammography, underscoring CEM's advantage in dense breasts. Panels (E) and (F): Contrast-enhanced CT chest images reveal enhancing soft-tissue densities in the left breast, corresponding to the lesions seen on mammography and CEM. No definite evidence of thoracic metastases is seen at this level. Panel (G): Histopathology (H&E stain, high-power view) from ultrasound-guided core biopsy demonstrates nests and cords of atypical epithelial cells forming glandular structures with desmoplastic stromal reaction—consistent with invasive ductal carcinoma (not otherwise specified). CEM: contrast-enhanced mammography.

A key strength of CEM lies in its ability to highlight angiogenic activity, thereby revealing additional malignant foci. In this study, CEM detected ≥2 additional lesions in 43.7% of patients, closely mirroring the histopathological prevalence of multiple malignant foci (43.2%). This was higher when compared with the results of the previous study of Di Grezia et al. and Taylor et al., which reported additional lesions in 13-15% of the patients [15,16]. The surgical impact was substantial, with 36.8% of patients requiring a change in surgical management, including conversion from breast-conserving surgery to mastectomy. This reinforces prior work by Goh et al. and Åhsberg et al., which demonstrated that CEM findings lead to clinically meaningful changes in surgical planning [17,18]. Figure 9 and Video 3 demonstrate a patient with type C dense breast in whom CEM detected an additional lesion, hence altering the surgical management.

**Video 3:** Digital breast tomosynthesis-guided visualization of primary lesion; however, multifocal disease is obscured by dense breast tissue. Digital breast tomosynthesis demonstrates heterogeneously dense parenchyma, limiting evaluation for small or deep lesions. A single irregular, spiculated mass is clearly visualized in the lower inner quadrant, showing associated architectural distortion—features suspicious for malignancy. However, no definite additional lesions are appreciable on DBT due to dense overlapping fibroglandular tissue. The multifocal disease seen on CEM remains obscured on both DBT and standard digital mammography, underscoring the superior lesion conspicuity and sensitivity of CEM in dense breast evaluation. DBT: digital breast tomosynthesis; CEM: contrast-enhanced mammography.

Accurate size estimation is pivotal for surgical planning and prognostication. DM and DBT both tended to overestimate lesion size compared to histopathology, consistent with reports by Azcona Sáenz et al. [19]. CEM, while also slightly overestimating by an average of 5 mm, demonstrated markedly better correlation with histopathology (r = 0.969-0.987, ICC >0.87). Bland-Altman analysis confirmed a narrow limit of agreement, reinforcing CEM as a reliable surrogate for histopathological size estimation. These findings are congruent with Sogani et al., who highlighted that CEM provides size estimations comparable to MRI [20] and superior to DM or DBT [21].

Radiologist confidence is critical in clinical practice, as equivocal interpretations can lead to unnecessary biopsies or missed diagnoses. In this study, 74.9% of CEM interpretations were rated at the highest confidence score, compared with only 18.8% for DM and 24.8% for DBT. This aligns with prior literature, which consistently shows higher lesion conspicuity and diagnostic certainty with CEM [22]. Enhanced conspicuity is especially valuable in dense breast populations where architectural distortions and subtle masses are otherwise obscured. The excellent inter-reader agreement for CEM, reflected by both high ICC and Cohen's kappa values, underscores its superior reproducibility and diagnostic consistency compared with DBT and DM, further strengthening its reliability as a frontline imaging tool in dense breasts.

Multivariate analysis revealed that CEM-detected additional lesions were the strongest independent predictor of surgical modification (OR ≈71.7), whereas lesions detected only on DBT paradoxically reduced the odds of surgical change. This highlights the unique clinical value of CEM: it not only detects more disease but does so in a way that meaningfully influences treatment. These findings are in line with a growing body of evidence that CEM can substitute for MRI in surgical planning when MRI is unavailable, contraindicated, or not cost-effective [23].

Only one-third of the patients had complete surgical follow-up, introducing potential selection bias and limiting generalizability. Although baseline features did not differ significantly between cases with and without surgical data, listwise deletion may have reduced statistical power and model stability. Future studies should incorporate multiple-imputation or inverse-probability weighting to mitigate missing-data bias.

While the benefits of CEM are increasingly recognized in Western populations, data from South Asia remain limited. Pakistan bears the highest breast cancer incidence in Asia, with a younger patient demographic and higher prevalence of dense breasts compared to Western cohorts. This study provides robust, region-specific evidence supporting the integration of CEM into routine diagnostic algorithms in dense-breast populations. While CEM demonstrated superior diagnostic performance and substantial influence on surgical planning, cost-effectiveness and MRI-replacement potential were not directly evaluated. CEM may represent a potentially accessible adjunct in settings where MRI is unavailable or contraindicated [16,24], but formal cost and resource analyses are required before policy-level conclusions.

In Pakistan, the implementation of contrast-enhanced mammography (CEM) remains limited, with just a handful of tertiary-care centers currently offering this modality. Moreover, local data indicate that more than half of the screened female population present with dense breast parenchyma (BI-RADS C or D), with one study reporting 62.4% of women in a tertiary center exhibiting dense tissue on mammography [25]. Given the high prevalence of dense breasts and the masking effect this poses for standard mammography, the wider availability and incorporation of CEM into diagnostic pathways is strongly advocated. In such a population, broader application of CEM could significantly improve lesion detection and preoperative staging, especially in centers where access to breast MRI is restricted.

This study has several limitations including: (1) A relatively modest sample size compared with multicenter trials; (2) Only 32.4% of patients had complete surgical records, introducing potential selection bias and limiting external validity; (3) The cohort was enriched for patients with multiple lesions, potentially inflating incremental detection compared with general dense-breast populations; (4) The single-center design and short accrual period may limit reproducibility; (5) Although imaging and reading protocols were standardized, absence of cross-modality randomization in the original design may have introduced minor interpretive bias; (6) Like MRI, CEM may miss small or low-vascularity lesions, and it is subject to field-of-view and artifact limitations [26,27].

Future multicenter studies with standardized acquisition parameters and complete surgical follow-up are warranted. However, despite these considerations, the findings strongly support the integration of CEM into breast cancer diagnostic pathways.

## Conclusions

In this enriched dense-breast cohort, CEM demonstrated higher sensitivity for multifocal and multicentric breast cancer detection and greater impact on surgical planning than DM or DBT. The results should, however, be interpreted with caution, given the limited sample size, single-center design, and lack of detailed protocol standardization. CEM emerges as a promising, potentially accessible adjunct for preoperative evaluation in dense breasts, pending validation through larger multicenter trials with full surgical correlation.
